# Strengths of community and health facilities based interventions in improving women and adolescents’ care seeking behaviors as approaches for reducing maternal mortality and improving birth outcome among low income communities of Egypt

**DOI:** 10.1186/s12913-020-05412-1

**Published:** 2020-06-29

**Authors:** Ammal M. Metwally, Ghada A. Abdel-Latif, Amira Mohsen, Lobna El Etreby, Dalia M. Elmosalami, Rehan M. Saleh, Marwa M. El-Sonbaty, Hala A. Amer, Sherif E. El Deeb, Asmaa M. Fathy, Carine Hanna, Osama Azmy, Tamer F. Taha, Amr Abbassy, Mahmoud Alalfy, Hatem Mohamed Hasan, Mohamed Abdelrahman

**Affiliations:** 1 Community Medicine Research Department, Medical Research Division, National Research Centre (ID: 60014618), P.O. 12622, Dokki, Giza, Egypt; 2 Child Health Department, Medical Research Division, National Research Centre (ID: 60014618), P.O. 12622, Dokki, Giza, Egypt; 3 Department of Pediatrics, College of Medicine, Taibah University, Madinah, Kingdom of Saudi Arabia; 4 Infection Control Department, King Saud Medical City, Riyadh, Kingdom of Saudi Arabia; 5 Reproductive Health Department, Medical Research Division, National Research Centre (ID: 60014618), P.O. 12622, Dokki, Giza, Egypt

**Keywords:** Educational interventions - care seeking behaviors, Emergency obstetric care- adolescent pregnancy- maternal mortality and birth outcome

## Abstract

**Background:**

Provision of emergency obstetric care is considered the key for maternal mortality reduction worldwide. This study evaluated the impact of community- and facility-based educational programs on provision of emergency obstetric care in Egypt. The study focused on evaluating utilization of the available health services and care seeking behaviors of mothers in the childbearing period.

**Methods:**

We implemented a package of community- and facility-focused educational interventions in two of Egypt’s lowest income governorates. At facility level, health professionals at rural health units from 21 villages over 5 years were trained. Mass media gathering, individual teaching at health facilities, printed materials and home-based care sessions were provided. Collectively, these interventions were designed to focusing on recognition of the early warning signs during pregnancy, delivery and postpartum period for timely referral to hospitals for 20,494 women and adolescents mothers.

**Results:**

The impact of the interventions was highly reflected on the percent of mothers received care during their pregnancy period. Proper antenatal care at governmental or private health facilities was raised dramatically from 0.6 to 59.3% and those who utilized at least one family planning method from 61.4 to 74.4%. Accordingly, the rate of complications significantly reduced during pregnancy (38.1 to 15.1%), during delivery (24.1 to 13.1%) and during postpartum (81.7 to 7.0%). As an impact to the improvement, there was a marked reduction in adolescent pregnancy by 55% and better birth outcome with a reduction in the percent of stillbirth by 11.5%.

**Conclusion:**

It is important to provide a comprehensive package that works at both improving qualities of care as well as empowering women by knowledge to first aid measures at the community level. The cost-effective way to empower mothers to provide first aid measures as emergency obstetric care is to adopt the outreach approach which could be more influential than mass media campaigns for the at-risk and vulnerable and low-income communities.

## Background

Improving maternal and child health is foundation for every nations, communities and families. Millennium Development Goals (MDGs) had applied various strategies to overcome the gaps resulted in maternal and child health morbidity and mortality from global to local levels [1]. Over the last 15 years, maternal and neonatal mortality has declined in most parts of the world, although not as much as anticipated when the MDGs were agreed to in 2000 [2].

Globally, there is a greater awareness of the complications associated with pregnancy, childbirth and/or postpartum and also birth outcome [3]. In February 2015, the World Health Organization (WHO) released strategies toward ending preventable maternal and neonatal mortality, outlining global targets and strategies for reducing maternal and neonatal mortality in the Sustainable Development Goal period for which, the health of mothers and children is central [4].

In low income communities, high maternal and neonatal mortality is driven partially by communities due to the limited access to facility-based obstetric care—particularly for emergencies.” Maternal and neonatal mortality ratio is still high because many mothers did not seek proper care in the health facility when they were exposed to any of pregnancy, childbirth and/or postpartum complications. They also did not receive proper ANC (4 visits), did not deliver by well-trained health providers, and did not seek PNC within 40 days after delivery [5].

In Egypt, there is clinic based studies targeted doctors and nurses only at the health facilities of the districts levels [6, 7]. Community based interventions that were done were limited to health education of small number of women in childbearing age and for short period of time [8]. These studies focused on general reproductive health issues without getting in depth for emergency obstetric care related to antenatal, natal and postnatal risks and complications. Moreover, these interventions revealed that more needs for training of health providers and improving the knowledge of women especially in low income areas.

Although, the majority of researchers indicated that most maternal deaths and neonatal problems are preventable if mothers receive essential and continuous healthcare services before, during, and after childbirth, yet the utilization of maternal health care services is still considerably low among both rural and urban mothers in general and adolescents’ mothers in particular [3, 9–12]. In Egypt, it is also well documented that empowerment of health providers to be more knowledgeable and skilled together with raising awareness of women in general and adolescents in particular to their reproductive rights are crucial to reduce maternal mortality through facilitating outreach activities, increasing access to medical facilities, and providing first aid measures for managing complications [13, 14]. Moreover, antenatal education interventions have an impact on the health of the pregnant woman and their children [15]. A mix of interventions at both community and health facilities for creating demand, provision of emergency obstetric services and empowering women and adolescents mothers with knowledge is indicated to make a difference for reducing maternal mortality and improving lives of communities.

This study broad goal was to contribute to the reduction of maternal mortality and reducing adverse pregnancy outcomes. The specific objectives aimed to evaluate the effect of a community and health facilities based intervention program on both the utilization of the available health services within governmental health facilities for women targeting health facilities and on care seeking behaviors of women in childbearing period and adolescents’ mothers. The study also measured the influence of the interventions on rate of complications and effect on birth outcome, and identified the most common channels through which interventions can improve care seeking behaviors among mothers. The implemented interventions included educational package directed to both health facility workers and to women in the childbearing period.

## Methods

### Study setting and design

This study was both community and facility based that was conducted in twenty one villages and 119 satellites of two governorates of Upper Egypt which were belonging to Al Fayoum and Benisuef governorates. The targeted districts and villages were remote areas with difficulty accessing health facilities by the villagers. Out of the selection criteria for the targeted villages, was that these areas were reported to have a very high rate of complication.

The study was an interventional evaluation study with before and after comparison. This study was conducted over 5 years. Prior to the intervention, the study randomly targeted 643 multiple pregnant mothers (primiparous mothers were excluded) within 500 households for the initial assessment phase; the interventions targeted 20,494 women within their prenatal, natal, and postnatal period along 5 years. After the intervention, the study randomly targeted 773 multiple pregnant mothers (primiparous mothers were excluded) within 500 households for the evaluation phase.

#### Phases of the study

- ⌧ The first phase included assessment of the level of beneficiaries’ behavior towards prenatal, natal and postnatal care on a sample of mothers during pregnancy, lactation and postpartum period [5].
- ⌧ The second phase of the study included conduction of community and health facilities based interventions for all of mothers during pregnancy, lactation and postpartum period (no = 20,494) along the 21 targeted villages. All the intervention that enables provision of components of essential obstetric care services at both the facility and community levels were conducted by trained health workers [13]. The interventions started in 2013 and has completed in 2018
- ⌧ **Mechanism of provision of the maternal interventions:**

1. Promotional educational materials were developed keeping in view the users and the respondents (beneficiaries) in the community (refer to Table 1). The messages of the materials focused on emergency care for the direct causes of maternal deaths, clinical criteria and management for the basic obstetric services with special emphasis on enhancing proper recording and registration of risk pregnancies and obstetric emergencies, and on improving quality of care.
2. All the developed and produced materials (four types of the promotional materials) were distributed to all targeted mothers; at health facilities, they were educational sessions and mass media and at the community level outreach activities with give away calendars.
3. Training and refresher courses were delivered to the primary health care physicians and nurses as it is the first level to be in contact with the pregnant woman at the governmental health facilities. Another group was the informal health providers as CHWs in MOH known as Raedat who were at the same time members of community based organizations (CBOs). The trained personnel were: 26 physicians and 103 nurses within health units, 63 Community health workers (CHWs) for providing the outreach campaigns and home visits. CHWs included Raedat and traditional birth attendants (TBAs).
4. Promoting culturally-competent behavioral changes through messages to the targeted mothers distributed by the previously well-trained health workers.

**Table 1:** Dissemination approaches for the educational messages

Activities	Responsibility	Supervisors	Target group	Tools and materials used
**Health facility based messages (weakly sessions)**	Physicians and nurses in the health facilities	Professional trainers from National Research Centre	Mothers in childbearing period with special focus on pregnant mothers in postpartum period.	Face to face educational sessions with power point, game who will win with us and the three wall posters for the prenatal, natal and postnatal care.
**At least eight outreach home visits for each mother; 4 during pregnancy; 4 during postnatal period**	Nurses and Raedat [community health workers (CHWs)]			Educational calendars with the key messages as a give away
**Mass media in the waiting halls within the health facilities**	Nurses			Film Salamtek

The CHWs were chosen because they are proved to play an important role in the life of mothers in the rural and urban low-income communities in Egypt [15–17]. Each CHW as “insider” play an integral part of the community in which they live and operate on a shared system of beliefs. They understand the community traditions, have the skills of communication and observation and are familiar with the housewives of their communities.

### The developed educational promotional materials used for awareness raising and public education

#### Description

Four types of the promotional materials included the following:

1. Calendar,
2. Three wall posters,
3. Game who will win with us (derived from who will win a million; who wants to be a millionaire) and
4. Drama production on CDs

#### Key topics/contents of the messages

1. The training for the physicians and nurses focused on level of care during antenatal, natal, and postnatal period; clinical emergency obstetric knowledge, clinician obstetric management skills. During the refresher courses the developed promotion materials were used as aiding materials to deepen and sustain the gained knowledge.
2. The theme of the developed messages to communities was what mothers and their families need to know regarding care according to the period of continuum of care, all the services that are provided within the primary health facilities and how to recognize danger sign and provide first aid measures to mitigate complications, where to go for delivery care and emergency care and how to arrange in advance for transport.

#### Direct beneficiaries

To women and adolescent females *(which is defined as females whose age from 10 to 19 years old, which constitutes 62% in the current study)* in childbearing period and mainly to illiterate one that constitute 47.7% [5] of the targeted mothers during the dissemination of the behavioral change messages with special focus on pregnant mothers and those in postpartum period.

- ⌧ **Evaluation Phase:** Evaluation of care seeking behavior of sample of mothers during pregnancy, lactation and postpartum period was done. The percentage of change in level of mothers behavior was conducted. Special emphasis was considered as regard indicators for action taken upon recognition of the early warning signs during pregnancy, delivery and postpartum period, timely referral to governmental, non-governmental and private hospitals.
- ⌧ To evaluate the most credible channels through which interventions can improve the care seeking behaviors of mothers, the targeted 500 households were divided equally into 4 types of media channels. The first quarter targeted 197 mothers who were exposed to all different types of interventions in 125 houses, the second quarter targeted 194 mothers who were exposed to educational sessions and mass media at facility level in 125 houses, the third quarter targeted 196 mothers who were exposed to outreach and give away calendar at community level in another 125 houses and the last quarter targeted 186 mothers who were not exposed to any types of interventions in the last 125 houses.

### Sample size and sampling technique during assessment and evaluation

The targeted districts and villages were selected randomly from remote areas out of the difficult to access health facilities by the villagers.

From these villages two-stage random sampling were used; households were randomly selected, and then one mother of reproductive age was randomly selected from each household who were either pregnant or in postpartum period.

A sample size of 494 households produces a two-sided 95% confidence interval with a width equal to 0.090 when the sample proportion is 0.500 [18]. This is rounded to 500 households having mothers of reproductive age were contacted.

Household lists having pregnant mothers in the postpartum period were obtained from CHWs zones within the targeted villages for which the CHWs were the interviewers.

The sample size calculation was based on the number of households which were 500 households prior to and after the interventions. Because of the fact that in rural areas, we accustomed to have extended families, so we obliged to include all women who fulfilled the eligibility criteria. Accordingly, All eligible women who had delivery during the intervention period and were living in the randomly selected households have been included for the survey; 643 prior to interventions with average 1.3 (either one or two women in each household) and 773 during evaluation phase with average 1.5 (either one or two women in each household).

#### Data collection types and tools

The data-collection instrument was through mothers’ interview (questionnaire). A structured questionnaire that was tailored from that of the CDC, 2010 was used [19], as well as from the information given to the pregnant mothers having the pregnancy card during their ANC visits which was designed and authorized by the Maternity and Childhood section in the Ministry of Health in Egypt. A structured interview was done with other women that did not include in the study prior to the assessment and before the start of the intervention types and was the base for the tailored questions that were added to the CDC questionnaire. It included questions related to mothers behavior about signs of obstetric complications (antenatal, natal, postnatal), complaining of any obstetric complication, what to do if the woman has any of these signs (refer to Table 4) and their utilization to the available health services within the governmental health facilities (refer to Table 3).

### Providers during the assessment and evaluation were:

- ⌧ *Not-Skilled/not-trained providers during the Initial Assessment and during the Evaluation included: mothers during pregnancy, lactation and postpartum period non trainded CHWs, Raedat, and TBAs.*
- ⌧ Skilled/trained providers during the Evaluation included: 63 CHWs (Raedat, and TBAs).
- ⌧ *Skilled/trained health providers during the Initial Assessment and during the Evaluation included: 103 nurses and 26 physicians.*

A pediatric consultant who has experience in dealing with adolescent and behavior shared and gave instructions during assessment, intervention and evaluation of adolescent females for better results

### Data management Analysis

All completed questionnaire forms were entered in the computer, Statistical Package of Social Science Software program (SPSS), version 16 to be statistically analyzed. Descriptive statistics such as frequency and percentage were used for data summarization. Diagrams and figures were used to illustrate simple information by using bar charts. The change in the practices were the basic instruments for comparison. The obtained data of each participant was collected and translated into quantitative ones and tabulated. The analysis was done using Z test between two proportions for all pre/post comparisons of all indicators [20]. *P* value < 0.05 was considered significant and P value < 0.01was considered highly significant.

#### Indicators used during the assessment and evaluation were

In order to measure the success in inducing the required changes in mothers’ behavior for issues related to maternal health, maternal mortality and better birth outcome, priority indicators were selected and measured in the selected districts twice; during the initial assessment and after the interventions with the aim of comparing the values of these indicators and calculating the percent change before and after the interventions.

We developed and used the following indicators:

**10 care seeking behavior indicators during antenatal, natal, and postnatal periods included** the percent of surveyed mothers

- **During pregnancy; who received:**
  1. *proper ANC visitsat governmental or privatehealth facilities*
  2. *at least one ANC visitat governmental or privatehealth facilities*
  3. *at least one ANC visit at home byskilled/trainedproviders*
- **During delivery; whose delivery was attended:**
  1. *at homebynot-skilled/not-trainedproviders*
  2. *at homebyskilled/trainedproviders*
  3. *at governmental or privatehealth facility*
- **During postpartum period; who:**
  1. *did not received proper postpartum care byskilled/trainedproviders*
  2. *received proper at home byskilled/trainedproviders*
  3. *received proper postnatal careat governmental or privatehealth facility*
  4. *have ever used at least one modern FP method*

**10 indicators of care seeking behavior out of mothers who reported antenatal, natal, postnatalcomplications included.** The complications were both self-reported ones as well as confirmed by records for those who attended the health facility within their villages

The indicators were: the percent of the mothers:

- **During pregnancy; who***reported pregnancy complications and***:**

1. *who not receiving the proper ANC at health facility*
2. *sought helpat homebynot-skilled/not-trainedproviders*
3. *sought helpat homebyskilled/trained providers*
4. *sought helpat governmental or privatehealth facilities*

- **During delivery;***who reported delivery complications and***:**

5. *sought help at home by not-skilled/not-trained providers*
6. *sought help at home by skilled/trained providers*
7. *sought help at governmental or private health facility*
8. *sought help at home by not-skilled/not-trained providers*

- **During postpartum period;***who reported postpartum complications and***:**

9. *sought help at home by skilled/trained providers*
10. *sought help at governmental or private health facility*

Percent change of adolescent pregnancy was also calculated. Percent change of birth outcome (abortion, stillbirth, low birthweight, birth defects … etc) was calculated, Babies were examined by pediatrician in the maternal and child health facility and checked by the current study pediatricians to detect any abnormalities.

### Ethical considerations

The study complied with the International Ethical Guidelines for Biomedical Research Involving Human Subjects [21]. The Research and Ethical Committee of the National Research Centre has cleared the study protocol with ethical approval registration number 10140. Informed consent was obtained from all participants involved in the study and information obtained at the individual level was kept strictly confidential.

## Results

As regard the impact of intervention education program on ANC, delivery care and PNC and their complications, Table 2 shows that there was highly significant difference regarding ANC and PNC between the initial assessment and the evaluation. It also shows that the percent of the maternal complications during pregnancy, delivery and postpartum had marvelous decline with highly significant difference from the initial assessment till the evaluation (*p* < .0001).

**Table 2:** Impact of Intervention Education Program on ANC, Delivery Care and PNC and their Complications

Characters	Initial Assessment No = 643		Evaluation No = 773		***P*** value
	No	%	No	%	
1. Received ANC (one ANC visit or more)	515	80.1%	690	89.3%	.0001**
2. Received natal care	458	71.2%	652	84.5%%	.0001**
3. Received PNC	266	41.4%	409	52.9%	.0001**
4. Exposed to pregnancy complications	245	38.1%	158	15.1%	.0001**
5. Exposed to delivery complications	155	24.1%	101	13.1%	.0001**
6. Exposed to postpartum complications	525	81.7%	54	7.0%	.0001**

☆ **Pregnancy complications**: complications during pregnancy (anemia, heavy bleeding, pre-eclampsia, eclampsia, abortion, ectopic pregnancy, obstructed labor, sepsis, severe abdominal pain, fainting, vomiting, premature rupture of membranes, convulsion, oedema, toxemia, stop movement, or severe headache). **Delivery complications**: complications during delivery (premature rupture of membranes, bleeding, prolonged labor, abnormal fetal presentation, bad oedor fluid, fever, or contraction > 8 h). **Postnatal complications**: complications during puerperium (puerperal sepsis, bleeding, convulsions, severe lower abdominal pain, fainting, or general weakness)

* = Sig, ** = highly sig

Concerning care seeking behavior indicators during antenatal, period, Table 3 shows that out of receiving ANC, the percentage of surveyed mothers who received proper ANC at either governmental or private health facilities showed an obvious improvement from 0.6 to 59.3% with highly significant percent change. At least one ANC received at home by skilled providers was also increased with highly significant percent change. While receiving less than 4 ANC visit at health facilities was significantly decreased from 99.4 to 35.6% (*p* < .0001). Regarding the desirable delivery care indicators, there was significant improvement (*p* < .0001) after the interventions for the majority of the indicators. As for the care seeking behavior indicators during postnatal periods, the percent changes of those who received proper postpartum care at home by skilled/trained providers increased from 6.0 to 24.7% (*p* < .0001). However, mothers who received postpartum care at home by non-skilled/non-trained providers decreased from 41.7 to 16.4% (*p* < .0001). The results also revealed improvement with highly significant percent change in the percentage of mothers who ever used at least one modern FP method (*p* < .0001) **(**Table 3**)**.

**Table 3:** Care seeking behavior indicators during antenatal, natal, and postnatal periods

10 ANC, Delivery Care and PNC Indicators☆	Initial Assessment No = 643		Evaluation No = 773		Percent Change	***P*** value
	No	%	No	%		
1. ANC received at facility	3	0.6% Out of 515	409	59.3% Out of 690	61.8%	.0001**
2. Less than 4 ANC received at facility	512	99.4% Out of 515	246	35.6% Out of 690	−61.8%	.0001**
3. At least one ANC received at home by skilled providers	0	0.0% Out of 515	35	5.1% Out of 690	−5.1%	.0001**
4. Delivery attended at home by non-skilled providers	185	28.8% Out of 458	121	15.7% Out of 652	−13.1%	.0001**
5. Delivery attended at home by skilled providers	91	14.1% Out of 458	193	25% Out of 652	10.9%	.0001**
6. Delivery attended at facility	367	57.1% Out of 458	459	59.3% Out of 652	2.2%	0.232
7. Postnatal care received at home by non-skilled providers	111	41.7% Out of 266	67	16.4% Out of 409	−25.3%	.0001**
8. Postnatal care received at home by skilled providers	16	6.0% Out of 266	101	24.7% Out of 409	18.7%	.0001**
9. Postnatal care received at facility	139	52.3% Out of 266	241	58.9% Out of 409	6.6%	.046*
10. Ever used at least one modern FP method	395	61.4% Out of 643	575	74.4% Out of 773	13%	.0001**

☆ **Antenatal care**: Mothers receiving proper ANC visits (4 visits or more) during pregnancy by skilled/trained providers at health facility and/or trained Raedat at home or as outreach by trained nurses. **Natal care:** All deliveries by a skilled/trained physicians or nurses or birth attendant either at institutional hospital or at home. **Postnatal care:** Proper care of the mother and baby in the first 6 weeks (40 days) after childbirth by skilled/trained providers at health facility or at home. First postnatal contact should be as early as possible within 24 h of birth and at least three additional postnatal contacts are recommended for all mothers and newborns, on day 3 (48–72 h) and between days 7–14 after birth, and 6 weeks after birth [5]**.**

* = Sig, ** = highly sig

On the other hand, Table 4 reveals improvement in many indicators of care seeking behavior out of mothers who reported antenatal, natal, postnatal complications. The percentage of mothers who gave a history of pregnancy complications without receiving the minimum ANC visits at health facility was dropped from 83.3 to 34.2% with highly significant percent change (*p* < .0001). Moreover, there were highly significant improvement concerning complications happened to studied mothers at home (*p* < .0001). In addition, the percentage of mothers receiving first aid measures for their postnatal complications at home by skilled/trained providers was significantly increased from 25.3 to 49.6%. However, the evaluation showed a highly significant drop from 35 to 0% for the mothers who reported postnatal complications and sought help at home by non-skilled/non-trained providers. Utilization of facility health services increased as regard antenatal, natal and postnatal complications by at least 30% its level at the initial assessment **(**Table 4**).**

**Table 4:** Indicators of care seeking behavior out of women and adolescents who reported antenatal, natal, postnatal complications

7 Indicators of antenatal, natal, and postnatal complications☆	Initial Assessment No = 643		Evaluation No = 773		Percent Change	***P*** value
	No	%	No	%		
1. Pregnancy complications and not receiving ANC	204	83.3% Out of 245	54	34.2% Out of 158	−24.7%	.0001**
2. Pregnancy complications and sought help at home by non-skilled providers	119	48.6% Out of 245	23	14.6% Out of 158	−34%	.0001**
3. Pregnancy complications and sought help at home by skilled providers	25	10.2% Out of 245	47	29.7% Out of 158	19.5%	.0001**
4. Pregnancy complications and sought help at facilities	101	41.2% Out of 245	88	55.7% Out of 158	14.5%	.002**
5. Delivery complications and sought help at home by non-skilled providers	62	40% Out of 155	0	0.0% Out of 101	−40%	.0001**
6. Delivery complications and sought help at home by skilled providers	43	27.7% Out of 155	66	55.3% Out of 101	27.6%	.0001**
7. Delivery complications and sought help at facilities	50	32.3% Out of 155	35	44.7% Out of 101	12.4	.022*
8. Postpartum complications and sought help at home by non-skilled providers	184	35% Out of 525	0	0.0% Out of 54	−35%	.0001**
9. Postpartum complications and sought help at home by skilled providers	133	25.3% Out of 525	16	49.6% Out of 54	24.3%	.0001**
10. Postpartum complications and sought help at facilities	208	39.7% Out of 525	38	50.4% Out of 54	10.7%	0.064

* = Sig, ** = highly sig

Regarding the effect of the intervention on birth outcome, Table 5 revealed improvement in many indicators of birth outcome; total reduction in stillbirth was 11.5%, total reduction in low birth weight babies and premature babies were 5.1 and 2.3% respectively.

**Table 5:** Effect of the intervention on percentage of adolescent pregnancy and birth outcome

Indicator+ (8 indicators)	Initial Assessment No = 643		Evaluation No = 773		Percent Change	***P*** value
	No	%	No	%		
1. Current age at most recent birth is < 20 years	85	62%	54	7.0%	−55.0%	.0001**
2. Current age at most recent birth is > 40 years	0	0.0%	1	0.1%	0.1%	.211
3. Pregnancies ended in still births are ≥1	115	17.9%	50	6.4%	−11.5%	.0001**
4. Pregnancies ended in abortion are ≥1	268	41.7%	154	19.9%	−21.8%	.0001**
5. Current pregnancy is unwanted	257	39.9%	194	25.1%	−14.8%	.0001**
6. Most recently delivered babies with low birth weight	44	6.8%	13	1.7%	−5.1%	.0001**
7. Most recently delivered babies are premature	18	2.8%	4	0.5%	−2.3%	.0001**
8. Most recently delivered babies have birth defects	3	0.5%	1	0.1%	−0.4%	.079

**+ newly married – non pregnant women as well as 1st pregnancy are excluded from the calculation**

* = Sig, ** = highly sig

**Fig. 1:**
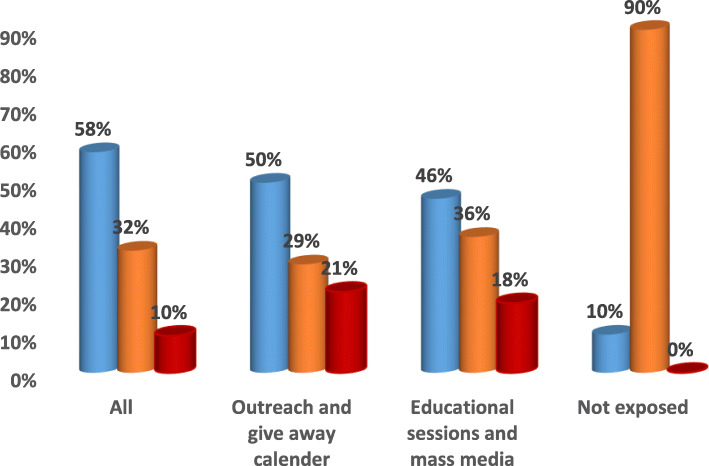
Channels of Intervention Education Program for ANC.  ANC received at facility.  Less than 4 ANC received at facility.  At least one ANC received at home by skilled providers

**Fig. 2:**
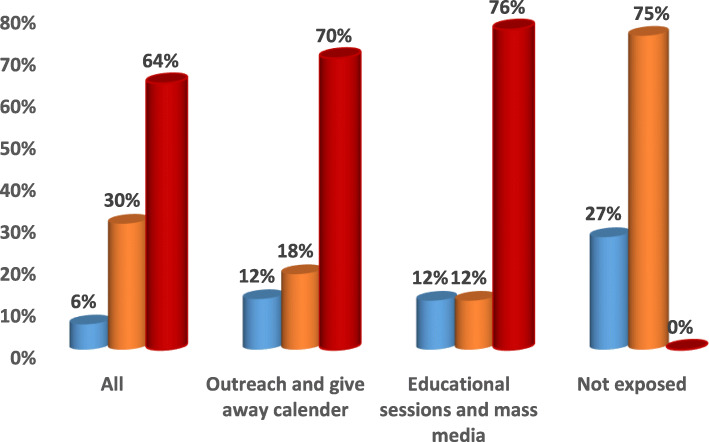
Channels of Intervention Education Program for Natal care.  Delivery attended at home by non-skilled providers.  Delivery attended at home by skilled providers.  Delivery attended at facility

**Fig. 3:**
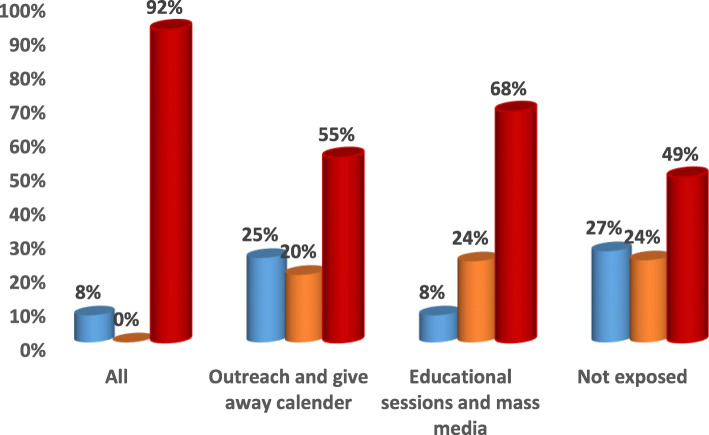
Channels of Intervention Education Program for PNC.  Postnatal care received at home by non-skilled providers.  Postnatal care received at home by skilled providers.  Postnatal care received at facility

**Fig. 4:**
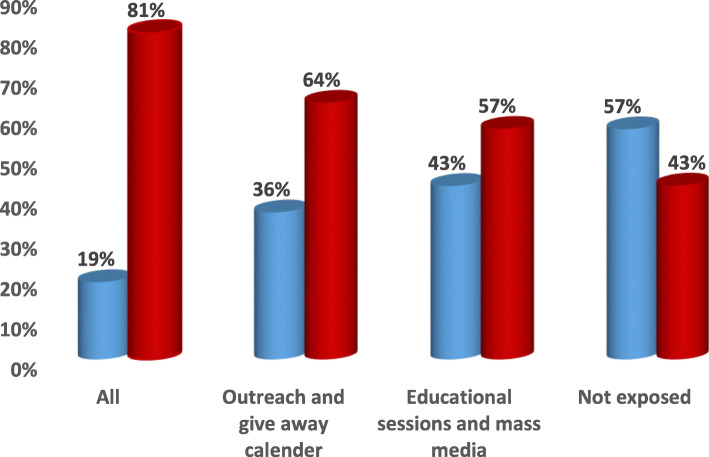
Channels of Intervention Education Program for Pregnancy Complications.  Pregnancy complications and sought help at home by skilled providers.  Pregnancy complications and sought help at facilities

**Fig. 5:**
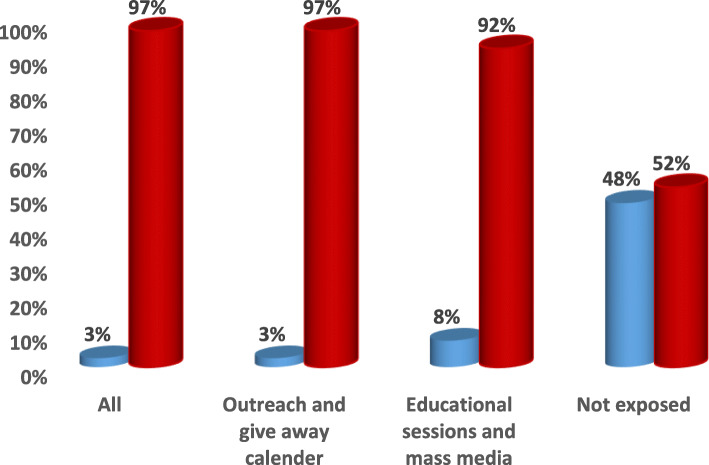
Channels of Intervention Education Program for Natal Complications.  Delivery complications and sought help at home by skilled providers.  Delivery complications and sought help at facilities

**Fig. 6:**
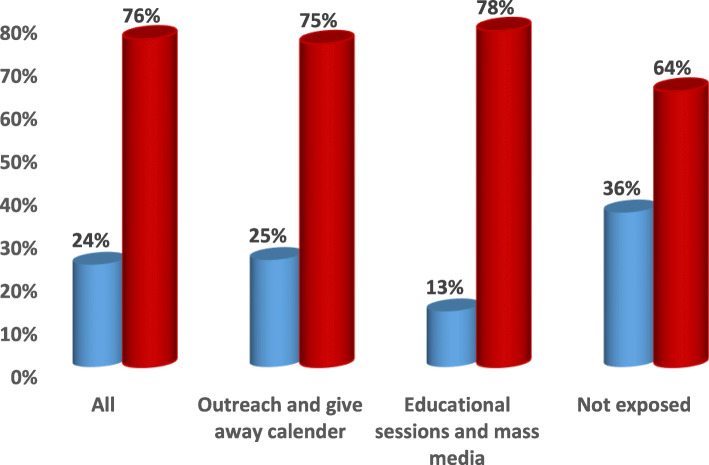
Channels of Intervention Education Program for Postnatal Complications.  Postpartum complications and sought help at home by skilled providers.  Postpartum complications and sought help at facilities

Combining all different types of intervention efforts at facility level (educational sessions and mass media) and community level (outreach and give away calendar) showed more values with highly significant difference when compared to each of them alone and to non-exposure in; increasing the awareness of the surveyed mothers and convince them to adopt healthy behaviors and in influencing the use of health care interventions related to antenatal, natal and postnatal periods and to the complications related to these periods (*p* < .0001) **(**Figs. 1, 2, 3, 4, 5 & 6**).**

## Discussion

The maternal mortality ratio (MMR) represents the risk associated with each pregnancy and it is defined as number of maternal deaths in a period per 100,000 live births [22]. Reduction in the global maternal mortality ratio (MMR) by 75% by 2015 was one of the MDGs targets. Despite numerous efforts, global MMR decreased by 44%. Meanwhile, in 2015, the MMR in developing countries was 239 per 100,000 live births compared to 12 per 100,000 live births in developed countries [23]. Improvement in maternal and neonatal care is one of the MDGs developed by the WHO [24]. This necessitates improvement of antenatal, natal and postnatal health care services [25, 26].

The change of MMR in our targeted governorates showed fluctuation with steady decline started during 2013 (with the beginning of our interventions) among the two governorates. The MMR was targeted by 2030 to be decreased to 30 and 35 deaths per 100,000 live births in El Fayoum and Benisuef respectively [27–29].

For care seeking behaviors during pregnancy, delivery and postnatal period, the percentage of mothers who sought care at home exceeded those at health facilities significantly. Also, the percentage of mothers who sought help for their complications and get managed at home by skilled health providers showed significant improvement as a result of the conducted interventions than those who sought help at health facilities with the minimum percent change of 19.5% versus 10.7% respectively prior and after the interventions. This is explained by the culture of those communities as mothers and their families still preferred to receive delivery care at home and they also trusted the health providers who received training courses during the study interventions.

Conventional means of education provision as brochures and mass media campaigns via television, radio and newspapers have become less effective in producing the desired behavioral changes in individuals especially for those in adolescent period, as 62% of our study group was adolescent females. Besides, these channels are considered to be relatively costly. The focus has been shifted to provide opportunities for people to learn skills in order to practice desired behaviors in their favored way of learning [30].

One of the aims of this study was to identify the most effective channels through which risky maternal behaviors could be changed to healthy ones and through which the utilization of the available health services could be increased. Results revealed that exposure to outreach and give- away calendars showed better results when compared to mass media campaigns and educational sessions within health facility regarding the percentages of care seeking behavior related to antenatal, natal and postnatal periods to prevent maternal deaths and poor birth outcome (stillbirth, low birth weight, birth defects). This may be due to the fact that the most of our study mothers was in the adolescent period (62%), illiteracy issues or possible mistrust in mass media rather than the outreach ones.

The present study aimed to evaluate the effect of the interventional education program on care seeking behaviors of mothers in childbearing period, to provide high quality care and to reduce maternal mortality and poor outcome. Maternal health interventions targeting health care workers have the potential to improve maternal and neonatal health services in the studied villages.

Our results showed that maternal health education exerts a significant influence on the utilization of maternal health facility services by mothers during pregnancy, delivery and postpartum period. Special emphasis was put on identifying mothers’ recognition of the early warning signs during pregnancy, delivery and puerperium, timely referral to hospital, and their utilization to the available health services within the governmental health facilities. Developed promotion materials were used as aiding materials to deepen and sustain the gained knowledge. It was also found that the percent of complications during pregnancy, delivery, postpartum and poor birth outcome (stillbirth, low birth weight, birth defects) showed an obvious decline from the initial assessment till the evaluation. ANC indicators showed significant improvement after the interventions. The percentage of surveyed mothers who received four or more antenatal visits from a formal providers at either the governmental or private health facilities increased significantly (59.2%).

Our study revealed total reduction in stillbirth was 11.5%. There is total reduction in low birth weight babies and premature babies 5.1 and 2.3% respectively. Total reduction in birth defects was 0.4%. Recent study showed that preterm labor, early neonatal death and LBW complication was reduced by 52, 61 and 46% respectively among women and adolescents with complete adherence to ANC visit [31].

Several studies discussed the importance of utilization of antenatal, natal and postnatal health care services especially education in lowering maternal mortality, morbidity and better birth outcome [32–34]. *Lassi and Bhutta (2015)* reported that maternal health education and home visits by trained community health workers can lead to significant reductions in maternal morbidity and neonatal mortality, and an increase in referrals to a health facility [35]. The positive influence of exposure to mass media on response of the mothers to utilize ANC services and to attending antenatal visits was obvious in many settings among various studies [13, 15, 16] than their non-exposed counterparts. Despite the known potential benefits of mass media campaigns, our results revealed that exposure to outreach and give away calendar showed slight better results when compared to mass media campaign and educational sessions within health facility regarding the percentages of care seeking behavior related to antenatal, natal and postnatal periods to prevent maternal deaths and neonatal problems. This was in accordance to a recent study for empowering rural women and adolescents in rural Egypt by raising their awareness through face-to-face interventions so that they became able to claim for their reproductive rights [17].

In recent decades, adolescent pregnancy has become an important health issue in a great number of developed and developing countries [36]. About 16 million adolescents aged 15–19 years and 2 million adolescents under the age of 15 years give birth every year, accounting for around 11% of all births. About 95% of these births occur in developing countries [37]. Previous study conducted in Upper Egypt proved that teenage mothers are more susceptible to preeclampsia, pre-term labor, premature rupture of membranes, oligohydramnios, vaginal delivery and their babies have lower birth weight and more neonatal reference to NICU and concluded that teenage parturient women are more likely to be housewives and resident in rural areas [38]. So this emphasizes the importance of awareness to reduce adolescent pregnancy and if happened reduce complications. In the current study, delivery care indicators showed that the percentage of women and adolescents whose delivery was attended at home by informal providers who used first aid measures was decreased significantly from 28.8 to 1.3% (*p* < 0.001). While, the percentage of women and adolescents whose delivery was attended by a formal providers at the governmental or private health facility was increased significantly from 57.1 to 67.3% (*p* < 0.001). Evaluation of postpartum care indicators in the present study revealed improvement in almost all selected indicators. The percentage of women and adolescents who ever used at least one modern FP method was increased significantly from 61.4 to 74.4% (*p* < 0.001). Also, the evaluation showed improvement concerning the percent change of women and adolescents who received postpartum care within 40 days after delivery at home by informal providers who use first aid measures (13.1%). A study in India reported that safe delivery and postnatal care were 53 and 40% respectively among rural adolescent ever married women who had any exposure of mass media, while women who did not have any mass media exposure were less likely to utilize safe delivery and postnatal care [39]**.** In accordance with other studies [30–41]**,** the present study has documented that maternal education has a significant impact on the utilization of maternal healthcare services among rural women and adolescents.

Our study revealed total percent change in adolescent pregnancy of 55.00%. Nearly one-fifth of adolescents become pregnant in Africa. Several sociodemographic factors like rural residence, ever married, not attending school of adolescents, low educated mother and father, were associated with adolescent pregnancy and complications. Interventions that target these factors are also important in reducing adolescent pregnancy and its complications [42]**.**

### Strengths of the study

Our study is characterized by being the first to provide interventions at both the micro (community) and meso (health facility) levels in Egypt. Our interventions aimed at first improving the quality of the health services provided at the health care facilities by the provision of training to the physicians and nurses within the targeted health facilities that led to increased utilization of the targeted women to the health services provided at the facility level. Second targeting the communities at their homes by the trained community health workers so that they become more knowledgeable to take care of themselves so that minimizing the risk of maternal complication.

The implemented interventions were designed primarily to prevent complications completely through care (“primary prevention”). Secondly, the interventions were directed to detect and mitigate the effect of the complications that might be happening so reducing the risk of maternal deaths (“secondary prevention”).

This study when providing a model for the comprehensive findings at both the micro and meso levels may have a great impact on communities with similar contexts.

### Limitation of the study

Although this study was community intervention along almost 5 years, it was limited to compare the impact of the interventions prior and after the implementation without having control group from similar communities that did not receive any intervention. In spite of the fact that this might be considered as a weakness of the study, yet including control group was difficult due to two reasons; first, the villages and their satellites that we have targeted were the worst ones for the availability of services along the two governorates with no matched other areas, second the ethical committee did not approve having control group without providing intervention to the control villages as a comparison group.

During the evaluation phase, because the rate of complication was low, we could not study the effect of any host or sociodemographic factors e.g. level of education, occupation … .etc. on different indicators as we could not fragment the data. This again might be considered out of the weakness of the study.

## Conclusion

Outreach and mass media educational campaigns proved to have profound effect regarding both the utilization of services as well as care seeking behaviors. This will definitely impact the reduction of maternal mortality and poor pregnancy outcome. It is important to provide a comprehensive package that works at both improving qualities of care as well as empowering people at the community level.

The most credible channel for communication to increase utilization of health facilities services and for women to be aware about their health is through reaching them. The cost effective way to convince mothers to adopt healthy behaviors is to adopt the outreach approach which could be more influential than mass media campaigns for the at risk and vulnerable and low income communities. So, it is recommended to avoid spending huge amount of money for mass media campaigns.

To bring positive behavioral change the awareness messages should be communicated effectively through well-designed messages and materials that are culture sensitive, age oriented and to be disseminated through capable disseminators who understand their communities.

## Data Availability

The datasets used and/or analysed during the current study are available from the corresponding author on reasonable request.

## Abbreviations

ANC: Antenatal care
CDC: Centers for Disease Control and Prevention
CBOs: Community based organizations
CHWs: Community health workers
FP: Family planning
MMR: Maternal mortality ratio
MDGs: Millennium Development Goals
PNC: Postnatal care
STDF: Science and Technology Development Fund
SPSS: Statistical Package of Social Science Software program
TBAs: Traditional birth attendants
WHO: World Health Organization
